# Difference in Background Parenchymal Enhancement on Contrast-Enhanced Mammography in Treatment-Naïve Versus Systemic Neoadjuvant Chemotherapy Patients

**DOI:** 10.7759/cureus.107590

**Published:** 2026-04-23

**Authors:** Oi Hang Hoon, San Yu Leung, Tsz Yan Ko, Kwun Man Mo, Yee Tak Lai

**Affiliations:** 1 Radiology, Pamela Youde Nethersole Eastern Hospital, Chai Wan, HKG

**Keywords:** background parenchymal enhancement, bpe, breast cancer imaging, cem, chemotherapy, contrast-enhanced mammography, neoadjuvant therapy

## Abstract

Background and purpose

Contrast-enhanced mammography (CEM) has emerged as a valuable imaging modality for breast cancer detection and management. Background parenchymal enhancement (BPE) reflects the level of normal glandular tissue enhancement and can vary based on physiological and pathological factors. Understanding the patterns of BPE in different patient populations, including post-treatment women, may provide insights into breast tissue response to therapy and aid in optimizing imaging interpretation. The purpose is to evaluate whether there is a difference in BPE on CEM in women who have undergone systemic neoadjuvant chemotherapy (NAC) to the breast compared with treatment-naïve women.

Methods

Retrospective single-center cohort study of consecutive women who underwent CEM from December 2019 to December 2024 inclusive. The degree of BPE, as defined by the American College of Radiology (ACR) Breast Imaging Reporting and Data System (BI-RADS) Atlas for each examination, was independently reviewed by two specialized breast radiologists, and interobserver agreement was evaluated using the kappa statistic (kappa score = 1.0 for both treatment-naïve and post-NAC groups) and categorized into minimal, mild, moderate, or marked. Treatment status was recorded. Cases that did not have paired data were excluded. SPSS version 29.0.0.0 (IBM SPSS Statistics for Windows, IBM Corp., Armonk, NY) was used for analysis.

Results

Among 132 studies, 45 cases had paired data. The background BPE showed no statistical difference between treatment-naïve and post-NAC groups. Treatment-naïve patients predominantly showed minimal or mild BPE (46.7% and 31.1%, respectively), and lower percentages of moderate to marked BPE (20% and 2.2%, respectively). Post-NAC patients demonstrated no statistically significant difference in BPE categories, with 42.2% showing minimal and 55.6% mild BPE. Moderate or marked BPE was infrequent (2.2% and 0%, respectively).

Conclusions

No statistically significant difference in BPE between treatment-naïve and post-NAC groups (p = 0.084).

## Introduction

Breast cancer remains one of the most commonly diagnosed cancers globally [1]. It is a notably heterogeneous disease with diverse histological patterns, which can be grouped into molecular subtypes: luminal-like, human epidermal growth factor receptor 2 (HER2)-enriched, and basal-like [2]. These subtypes are defined based on the expression of specific tumor biomarkers such as estrogen receptor (ER), progesterone receptor (PR), and HER2/neu overexpression. Each breast cancer molecular subtype differs in terms of incidence, how the disease progresses, survival outcomes, and how it responds to treatment, which are critical for guiding therapy choices and prognostic assessments.

Contrast-enhanced mammography (CEM) is an imaging technique that employs dual-energy X-ray and iodinated contrast agents to visualize the structural and anatomical features of breast tumors. It also highlights tumor neovascularity, similar to breast MRI [3]. Previous research indicates that CEM offers diagnostic accuracy comparable to MRI, with studies showing non-inferior performance [4]. Additionally, CEM is more accessible and cost-effective than MRI, making it a practical option in many clinical settings. One key feature observed in CEM is background parenchymal enhancement (BPE), which refers to the degree of normal fibroglandular breast tissue enhancement following contrast administration. Multiple factors, including age, menstrual or menopausal status, can affect breast glandular tissue enhancements [2-5].

Importantly, BPE may also reflect biological changes in breast tissue related to treatment effects, such as those induced by systemic neoadjuvant chemotherapy (NAC). However, how NAC impacts BPE on CEM remains incompletely understood.

BPE on breast MRI has emerged as an important imaging biomarker, with higher levels associated with increased breast cancer risk (OR 1.74-2.1) [6,7], hormonal/vascular activity in fibroglandular tissue, and potentially worse prognosis [8]. Studies also demonstrate that BPE reduction during NAC or endocrine therapy correlates with improved treatment response and lower recurrence risk [8]. However, these findings predominantly derive from MRI, while BPE behavior on CEM remains underexplored [9], providing the rationale for our study.

The primary objective of this study was to compare BPE on CEM between treatment-naïve patients and those who have undergone NAC. By elucidating differences in BPE, the findings may contribute to improved diagnostic accuracy and personalized imaging strategies in breast cancer management.

The null hypothesis is that the median difference of BPE between the treatment-naïve group and the post-NAC group is zero. The alternative hypothesis is that there is a statistical difference in the median difference in BPE between the treatment-naïve group and the post-NAC group.

This article was previously presented as a poster at the 24th International Cancer Imaging Society Meeting and Annual Teaching Course (24th-26th September 2025).

## Materials and methods

A retrospective single-center cohort study was performed, which included consecutive women who underwent CEM in a tertiary regional hospital between December 2019 and December 2024, inclusive. The patient cohort was identified through the institutional imaging database, and clinical data were extracted from electronic health records. Approval from the Hospital Authority Institutional Review Board was obtained, and individual consent for this retrospective analysis was waived.

Patients

**Figure 1:**
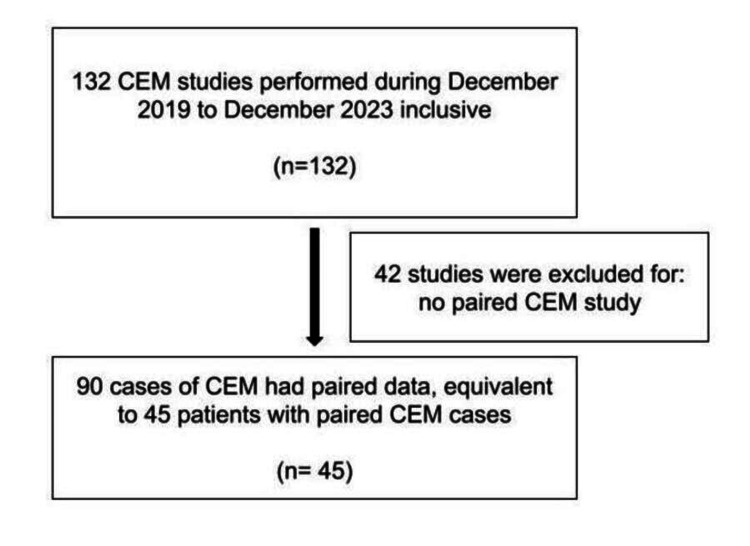
Patient inclusion and exclusion workflow CEM: contrast-enhanced mammography

A retrospective review of departmental cases with CEM performed from December 2019 to December 2024 inclusive retrieved from the local database was conducted. Information about the patients’ age, tumor biomarkers, and NAC used was also retrieved from the local database. Inclusion criteria comprised all women who received CEM for breast cancer screening, diagnosis, or treatment evaluation within the specified timeframe. Exclusion criteria included patients who had unpaired data (i.e., only one CEM performed) during the study period (Figure 1).

Each patient received NAC due to a large tumor size or with clinically documented lymph node involvement. Each patient received two MRI scans during this time period, one for baseline assessment prior to NAC, and a follow-up mid-cycle.

CEM examination

**Figure 2:**
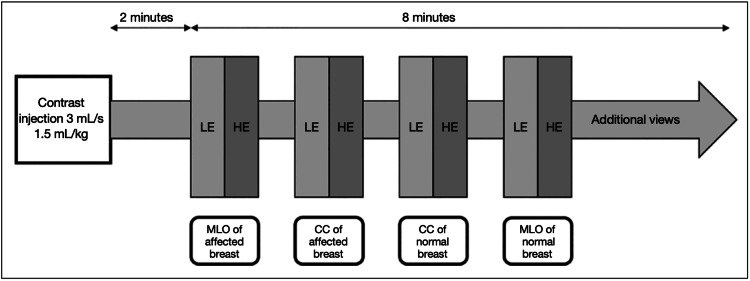
Contrast-enhanced mammography workflow CC: craniocaudal view; HE: high-energy; LE: low-energy; MLO: mediolateral oblique view Image made using Canva (Canva Inc., San Francisco, CA).

A CEM examination was performed utilizing the Hologic Selenia Dimensions Mammography System (Hologic, Inc., Marlborough, MA). The CEM contrast agent used was OmnipaqueTM (iohexol, GE Healthcare, Chicago, IL). The CEM images were viewed on high-resolution displays (5- to 12-megapixel (MP) monitors) for diagnostic review of mammograms. The volume of contrast administered is 1.5 mL/kg of the patient’s body weight at a rate of 3 mL/second through a power injector. CEM images were then acquired two minutes after contrast injection and completed within ten minutes. CEM low- and high-energy images in standard craniocaudal (CC) and mediolateral oblique (MLO) projections of each breast were obtained (Figure 2). CEM high-energy images were used in post-processing to produce the recombined images that showed areas of contrast enhancement. Only the low-energy and recombined images were displayed for review by radiologists. Each CEM examination was reviewed immediately by the session radiologist. Additional views (e.g., compression or magnified view) could be obtained if needed after reviewing the images. The time required for each CEM examination was recorded, starting at contrast injection and concluding at the last image obtained. 

Image interpretation and data entry

Analyzed imaging features included the presence of microcalcifications or architectural distortion on low-energy images and non-mass enhancement (NME) on recombined images. For low-energy images showing enhancement on recombined images, the assessment focused on mass shape and margins, internal enhancement patterns, extent of enhancement, and lesion conspicuity. BPE for all 90 CEM examinations was independently reviewed by two specialized breast radiologists with more than two years of experience in CEM interpretation in accordance with the American College of Radiology Breast Imaging Reporting and Data System (BI-RADS) lexicon for CEM, as published in 2022, and interobserver agreement was evaluated using the kappa statistic (kappa score = 1.0 for both treatment-naïve and post-NAC groups).

BPE was qualitatively graded into four categories: minimal, mild, moderate, or marked, based on the extent and intensity of normal fibroglandular tissue enhancement relative to background tissue. For patients with asymmetrical BPE, the side with the higher degree of BPE was used for data entry and analysis. It is of note that asymmetric BPE may arise naturally, but could also arise from differences in fibroglandular tissue volume or prior treatments such as breast-conserving therapy and radiation, which may reduce BPE on the treated side. As the study’s null hypothesis assumes no difference in BPE between treatment groups, analyzing the side with the higher BPE better captures the maximal enhancement and reduces the risk of underestimating BPE in patients affected by unilateral treatment effects or natural asymmetry.

Patient systemic treatment status was recorded from medical records and categorized into those who had undergone systemic NAC to the breast and treatment-naïve patients who had not received prior systemic therapy. Additional clinical variables, such as age, ethnicity, breast density, BPE symmetry, and whether there was a breast mass reported (if yes, the degree of lesion conspicuity), were collected to contextualize BPE findings.

Statistical analysis

All statistical analyses were performed using SPSS Statistics version 29.0.0.0 (IBM SPSS Statistics for Windows, IBM Corp., Armonk, NY). BPE distributions were classified as ordinal data (1 = minimal, 2 = mild, 3 = moderate, 4 = marked). Comparison of the paired data was conducted using the Wilcoxon signed-rank test for the treatment-naïve and post-NAC group. Statistical significance was defined as p < 0.05. 

The patients were further separated into subgroups of 1) pre-/peri-menopausal (<55 years old) versus post-menopausal groups (≥55 years old), 2) tumor biomarker, and 3) neoadjuvant regime. The BPE in baseline and post-NAC were again compared using the Mann-Whitney U test for each subgroup. p < 0.05 was considered significant.

## Results

Patient demographics are displayed in Table 1.

**Table 1:** Patient demographics table ER +ve: estrogen receptor-positive; ER -ve: estrogen receptor-negative; HER2 +ve: human epidermal growth factor receptor 2-positive; HER2 -ve: human epidermal growth factor receptor 2-negative

Patient characteristic		Number of patients
Total number of subjects		45
Age of patients		28-73 (mean ± SD 54.6)
Pre-menopausal		10
Post-menopausal		35
Ethnicity	East Asian	30
	Pacific islander	14
	South Asian	1
Tumor biomarker	ER +ve	23
	ER -ve	22
	Her-2 +ve	22
	Her-2 -ve	23
	Triple negative	9

According to the local institute’s Department of Oncology guidelines (Table 2), NAC is indicated in patients with HER2-positive or triple-negative breast cancer who have tumors ≥T2 or node-positive disease. For those with ER-positive and HER2-negative disease, indications include tumors ≥T3 or nodal stage N2. Relative indications include ER-positive, HER2-negative tumors with N1 disease, patients with a strong preference for breast-conserving therapy, and cases of locally advanced breast cancer with limited oligometastatic disease where individualized treatment may be appropriate.

**Table 2:** Neoadjuvant guidelines according to the local institute Department of Oncology guidelines ddEC: dose-dense epirubicin and cyclophosphamide; ER +ve: estrogen receptor-positive; HER2 +ve: human epidermal growth factor receptor 2-positive; HER2 -ve: human epidermal growth factor receptor 2-negative; TJH ± P: docetaxel, carboplatin, trastuzumab ± pertuzumab; P: paclitaxel

Tumor biomarker	Neoadjuvant chemotherapy treatment
HER2 +ve	Induction TJH ± P × 6 cycles
ER +ve, HER2 -ve	Induction ddEC × 4 cycles → P × 4 cycles
Triple -ve (immunotherapy not considered)	
Triple -ve (immunotherapy considered)	Pembrolizumab 200g q3w + weekly paclitaxel/carboplatin × 12 → EC q3w × 4 cycles

There is no statistical significance of pre- and post-neoadjuvant BPE between the two different neoadjuvant regimes (Table 3).

**Table 3:** Summary table of results BPE: background parenchymal enhancement; ddEC: dose-dense epirubicin and cyclophosphamide; ER +ve: estrogen receptor-positive; ER -ve: estrogen receptor-negative; HER2 +ve: human epidermal growth factor receptor 2-positive; HER2 -ve: human epidermal growth factor receptor 2-negative; NAC, neoadjuvant chemotherapy; P: paclitaxel; TJH ± P: docetaxel, carboplatin, trastuzumab ± pertuzumab

Category		Treatment-naïve BPE				Total (N)	Post-NAC BPE				Total (N)	Z score	p-value (p > 0.05)
		Minimal	Mild	Moderate	Marked		Minimal	Mild	Moderate	Marked			
Overall		21	14	9	1	45	19	25	1	0	45	-1.3798	0.084
Menopausal status	Pre-menopause	9	6	7	1	23	10	12	1	0	23	-1.2522	0.105
	Menopaused	12	8	2	0	22	9	13	0	0	22	-0.4695	0.319
Neoadjuvant regime	TJH ± P × 6	5	8	4	0	17	3	13	1	0	17	-0.1722	0.432
	ddEC × 4 + P × 4	5	2	2	0	9	4	5	0	0	9	-0.0442	0.482
	Pembrolizumab 200 mg every three weeks + weekly paclitaxel and carboplatin for 12 weeks, + EC every three weeks for four cycles	1	0	0	0	1	1	0	0	0	1	N/A	N/A
Tumor biomarker	ER +ve	9	1	1	1	12	9	3	0	0	12	-0.1732	0.431
	ER -ve	9	8	5	0	22	8	13	1	0	22	-0.4108	0.341
	HER-2 +ve	3	5	4	0	12	3	8	1	0	12	-0.7794	0.218
	HER-2 -ve	14	5	3	1	23	14	9	0	0	23	-0.3954	0.346
	Triple negative	5	3	1	0	9	5	3	1	0	9	0.000	0.500

The change of BPE in relation to different tumor biomarkers showed no statistically significant differences.

Statistical analysis using the Wilcoxon signed-rank test confirmed that the difference in BPE distribution between treatment-naïve and post-NAC women was not significant (z = -1.3798, p = 0.084), indicating no significant difference in the degree of BPE following systemic chemotherapy.

The baseline BPE was not statistically different between the pre-menopausal and post-menopausal groups (z = -1.2522, p = 0.105). The post-neoadjuvant BPE was also not statistically different between the pre-menopausal and post-menopausal groups (z = -0.4695, p = 0.319)

**Figure 3:**
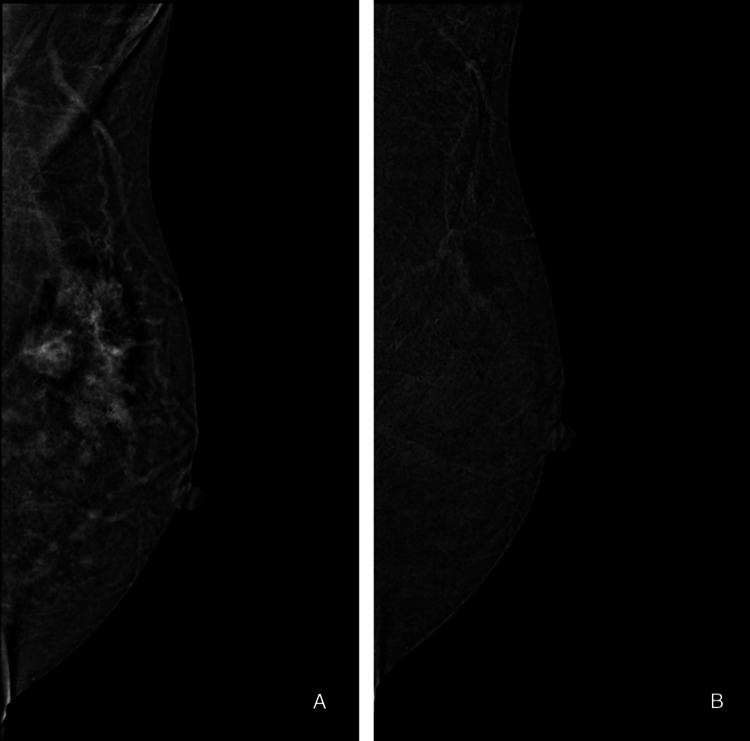
A case of left breast invasive ductal carcinoma in a 54-year-old woman (A) Treatment-naïve with moderate breast background parenchymal enhancement (BPE). (B) Post-neoadjuvant therapy with minimal breast BPE.

**Figure 4:**
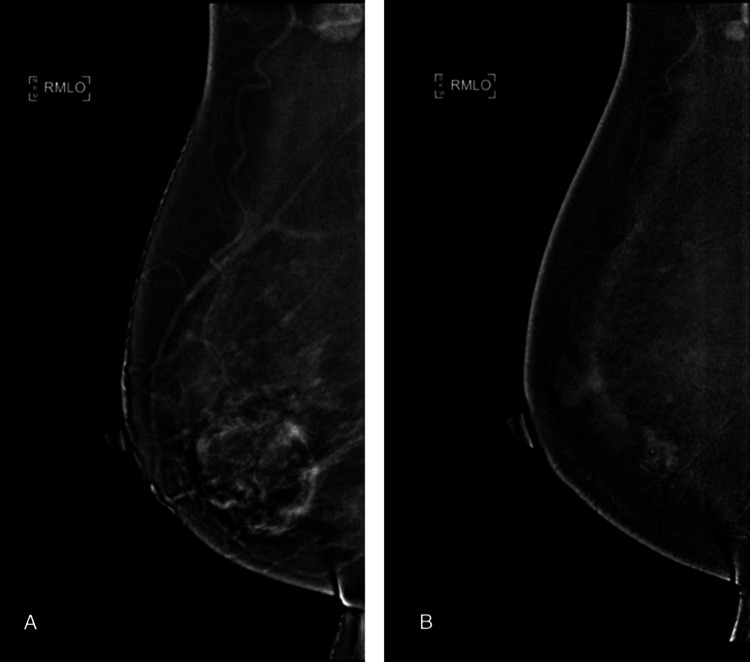
A case of right breast invasive carcinoma in a 52-year-old woman (A) Treatment-naïve and (B) post-neoadjuvant therapy contrast-enhanced mammography (CEM) show no significant difference in background parenchymal enhancement (BPE), both mild BPE.

**Figure 5:**
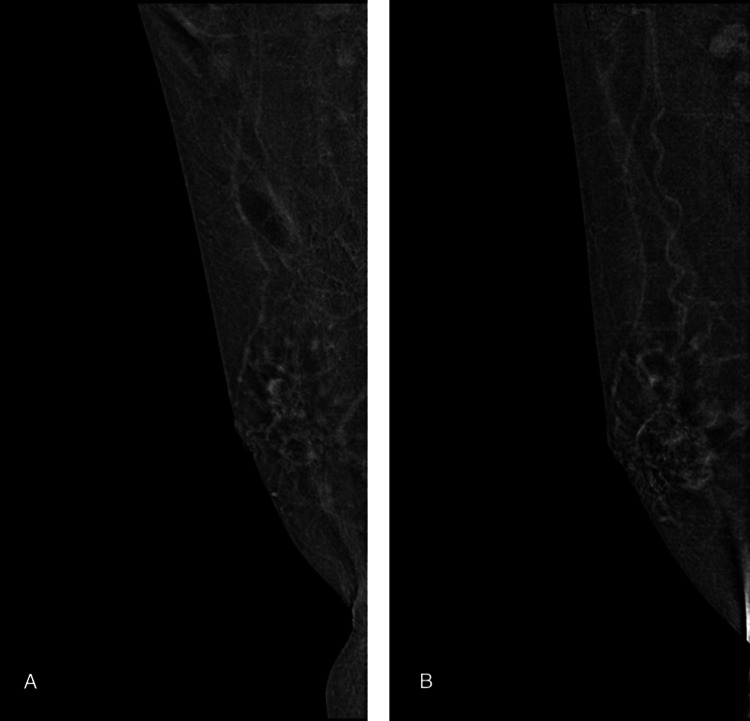
A case of right breast invasive ductal carcinoma in a 59-year-old woman (A) Treatment-naïve and (B) post-neoadjuvant therapy contrast-enhanced mammography (CEM) show no significant difference in background parenchymal enhancement (BPE), both moderate BPE.

Figures 3-5 illustrate representative cases of CEM images. 

## Discussion

Subgroup analyses are important to determine if the treatment effects observed in the overall cohort are homogeneous or if they vary among different patient populations based on clinical factors such as menopausal status, tumor markers, or treatment regimen. By demonstrating that there is no significant difference in BPE changes pre- and post-treatment within these subgroups, we ascertain the generalizability and consistency of the overall treatment effect. This approach helps to identify any potential effect modifiers and ensures that conclusions drawn from the overall analysis are applicable to diverse clinical subpopulations.

In this study of 132 patients, including 45 with paired pre- and post-NAC data, no statistically significant difference in BPE was observed between treatment-naïve and post-NAC groups on CEM. The majority of treatment-naïve patients demonstrated minimal or mild background enhancement, with only a small proportion showing moderate or marked BPE. Similarly, post-NAC patients predominantly exhibited minimal or mild enhancement, with virtually no cases of marked BPE. These findings suggest that systemic chemotherapy does not result in a measurable alteration of BPE on CEM, at least within the limitations of the current dataset. While previous studies have extensively examined changes in BPE in breast MRI after NAC, often associating reductions in BPE with treatment response or hormonal effects [5,10-15], our CEM results diverge markedly. Kim et al. [16] reported MRI BPE suppression in 78% of cases post-NAC, with subtype-specific differences (HER2+: 85% reduction; ER+: 62%), attributing changes to anti-angiogenic effects. Preibsch et al. [17] similarly found 71% BPE decrease, predicting recurrence-free survival. However, there is currently no published evidence describing pre- and post-NAC differences in BPE specifically assessed by CEM. Given that CEM and MRI captured different aspects of tissue vascularity and contrast kinetics, the absence of a significant difference in our study may reflect inherent modality differences or a relatively limited sensitivity of CEM for subtle background parenchymal changes [18,19]. Our null findings may thus reflect modality-specific sensitivity limits for subtle parenchymal shifts, potentially limiting their utility as an early imaging biomarker of response. 

While MRI BPE correlates inversely with age [20], no significant associations were found between BPE and age/menopausal status, indicating that BPE on CEM is not significantly influenced by baseline demographic or tumor biological characteristics in this cohort. In contrast, breast MRI literature has shown menopausal status as a key modulator of BPE, with premenopausal women exhibiting higher baseline BPE and greater post-treatment suppression due to hormonal fluctuations and chemotherapy-induced ovarian ablation [21]. Preibsch et al. [17] also showed premenopausal cohorts have 1.5- to two-fold higher minimal/mild BPE rates post-NAC compared to postmenopausal groups. Our CEM results diverge, potentially due to modality-specific contrast kinetics less sensitive to hormonal or receptor-driven vascularity. 

Tumor markers showed no association with BPE on CEM (ER/PR/HER2 status, all p > 0.10). HER2 status was determined according to current American Society of Clinical Oncology (ASCO)-College of American Pathologists guidelines [22], yet failed to correlate with BPE changes pre- or post-NAC. This contrasts with MRI literature, where baseline BPE predicts pathologic complete response (pCR) throughout NAC across subtypes [23], and HER2+ tumors show greater suppression (85% reduction) than ER+ cases (62%) [16]. Our CEM homogeneity across ER/PR/HER2-defined subgroups suggests this modality captures neither receptor-driven vascularity nor treatment response patterns observed on MRI.

No statistically significant differences in BPE were observed between pre- and post-NAC between the two standardized regimens in this cohort: 1) TJH (docetaxel, carboplatin, trastuzumab) ± P (pertuzumab) × 6 and 2) ddEC (dose-dense epirubicin and cyclophosphamide) × 4 + P × 4. These null findings align with limited MRI evidence on regimen-specific effects, where anthracycline-taxane sequences like ddEC + P (paclitaxel) show modest BPE reductions (mean 0.6 categories) in larger cohorts [10]. Contributing factors may include limited statistical power from small subgroup sizes, heterogeneity in patient menopausal status or hormone receptor profiles, and variability in imaging intervals post-treatment. 

The absence of BPE modulation by tumor biology, demographics, or regimens positions CEM BPE as a stable baseline metric rather than a dynamic NAC biomarker, unlike MRI, which affirms response predictivity [24]. Regional validation supports CEM's adjunctive role in Hong Kong practice [25], particularly when MRI is unavailable, though our findings caution against parenchymal-based response monitoring pending larger validation studies. Future investigations should prioritize larger, prospective cohorts with standardized regimens and serial imaging to delineate regimen-specific effects on BPE and correlate changes with pathologic response.

From a clinical perspective, these findings suggest that BPE in CEM may have limited utility as an imaging biomarker for treatment response or residual disease. Therefore, caution is warranted when extrapolating MRI-based interpretations of BPE to CEM. At the same time, the relative stability of BPE across treatment states observed in our cohort may be advantageous, as it allows for more consistent lesion conspicuity and longitudinal comparison. These findings help to better define the role and limitations of BPE in CEM and highlight the need for further investigation into its biological and clinical significance.

Limitations

The study has several limitations that should be acknowledged. Firstly, the sample size was relatively small, particularly for paired analyses, which may reduce the statistical power to detect small but clinically relevant differences. Future multi-institutional studies with larger cohorts are therefore warranted. In addition, as a retrospective single-center study, selection bias may have been introduced through clinical imaging indications and patient referral patterns. The findings may have limited generalizability to other populations, particularly those with different ethnic compositions, healthcare systems, or NAC protocols.

Secondly, several clinical variables that may influence BPE - such as menstrual status, use of hormonal therapy, and the interval between completion of NAC and imaging - were not fully standardized due to the retrospective study design. In particular, variability in the timing of post-NAC imaging may have introduced heterogeneity in BPE assessment. Although these factors were recorded where available, residual confounding cannot be excluded. Incomplete standardization of certain clinical variables and limited control of potential confounders may limit exact reproducibility across institutions, reflecting the inherent constraints of this retrospective study design.

Thirdly, without histopathological correlation or longitudinal follow-up, it remains uncertain whether post-treatment BPE changes are associated with therapeutic response or underlying tumor biology.

Additionally, the CEM images in this study were independently reviewed by two specialized breast radiologists, and interobserver agreement was evaluated using the kappa statistic. While the use of two readers is consistent with prior imaging studies, inclusion of additional readers may further strengthen reproducibility. As with MRI BI-RADS BPE assessment, visual scoring of CEM BPE remains subjective. Previous studies exploring the consistency of BPE evaluation between different readers have reported agreement levels ranging from fair to substantial, although these studies were conducted using MRI breast studies and not CEM. For example, Preibsch et al. [17] conducted an early pilot study involving 73 women undergoing preoperative breast MRI, where two independent readers evaluated BPE and found a fair level of agreement (weighted κ = 0.37). Additionally, Preibsch et al. [17] focused on BPE assessment in breast MRI before and after NAC, where they observed substantial agreement prior to treatment (κ between 0.73 and 0.77) that dropped to moderate agreement following therapy (κ ranging from 0.43 to 0.60). These findings emphasize that while some variability is inherent in qualitative BPE evaluation, clinical context may influence consistency.

Furthermore, there were cases that commented to have asymmetrical BPE, but an overall impression was used as the final BPE evaluation. This approach may reduce the accuracy and specificity of BPE characterization, as asymmetry can be clinically relevant, especially in identifying localized pathology. Additionally, collapsing heterogeneous BPE findings into a single overall score could increase variability and decrease reproducibility, and is again subject to reader interpretation. Lastly, as the study cohort consisted predominantly of East Asian, South Asian, and Pacific Islander patients, the findings may not be directly generalizable to populations with different ethnic compositions. Readers should therefore exercise caution when extrapolating these results to other regions, particularly where genetic backgrounds, environmental exposures, or patterns of healthcare delivery differ substantially from those in our study setting. 

## Conclusions

In this cohort of women who imaged with CEM, post-NAC women showed no statistically significant difference in BPE (p = 0.084) compared to pre-NAC. These findings suggest that, in this sample, NAC did not produce a measurable change in BPE that reached conventional levels of statistical significance, although a trend toward difference cannot be ruled out. Further studies with larger samples, standardized imaging intervals, and comparison across imaging modalities are warranted to clarify whether NAC has any measurable effect on BPE in CEM and to validate potential clinical implications.
